# Metabolic Seizures

**DOI:** 10.3389/fneur.2021.640371

**Published:** 2021-07-06

**Authors:** Mohammed Almannai, Rabah A. Al Mahmoud, Mohammed Mekki, Ayman W. El-Hattab

**Affiliations:** ^1^Section of Medical Genetics, Children's Hospital, King Fahad Medical City, Riyadh, Saudi Arabia; ^2^Department of Clinical Sciences, College of Medicine, University of Sharjah, Sharjah, United Arab Emirates; ^3^Department of Pediatrics, University Hospital Sharjah, Sharjah, United Arab Emirates; ^4^Department of Pediatrics, Al Qassimi Women's and Children's Hospital, Sharjah, United Arab Emirates; ^5^Clinical Genetics, University Hospital Sharjah, Sharjah, United Arab Emirates

**Keywords:** epilepsy, seizures, metabolic diseases, inborn errors of metabolism, mitochondrial diseases

## Abstract

Metabolic diseases should always be considered when evaluating children presenting with seizures. This is because many metabolic disorders are potentially treatable and seizure control can be achieved when these diseases are appropriately treated. Seizures caused by underlying metabolic diseases (metabolic seizures) should be particularly considered in unexplained neonatal seizures, refractory seizures, seizures related to fasting or food intake, seizures associated with other systemic or neurologic features, parental consanguinity, and family history of epilepsy. Metabolic seizures can be caused by various amino acids metabolic disorders, disorders of energy metabolism, cofactor-related metabolic diseases, purine and pyrimidine metabolic diseases, congenital disorders of glycosylation, and lysosomal and peroxisomal disorders. Diagnosing metabolic seizures without delay is essential because the immediate initiation of appropriate therapy for many metabolic diseases can prevent or minimize complications.

## Introduction

Inborn errors of metabolism (metabolic disorders) are relatively uncommon causes for pediatric seizures; nevertheless, they should always be considered when evaluating children presenting with seizures. This is because many metabolic disorders are potentially treatable and seizure control can be achieved when these diseases are appropriately treated (1–4). The immediate initiation of appropriate therapy for many metabolic diseases can prevent or minimize complications; therefore, the recognition of these disorders in a timely manner is of utmost importance. Even when dealing with metabolic disorders for which effective treatment is not yet available; making the diagnosis is very important to direct management plans and provide appropriate counseling related to prognosis and recurrence risk and prevention (1–4).

More than 200 different metabolic diseases are known to cause seizures. In these metabolic diseases epilepsy can be the main presenting manifestation or part of complex phenotypes with other neurological and metabolic manifestations (4). Metabolic diseases can cause seizures by different mechanisms. Seizures can occur due to the accumulation of toxic metabolites such as ammonia. Hyperammonemia occurs in several metabolic diseases such as disorders of the urea cycle and organic acidemias. Ammonia accumulation is neurotoxic as it results in increased glutamine synthesis causing swelling of astrocytes and brain edema (5). Some metabolic disorders can disturb neurotransmission. Glycine is an NMDA (N-methyl D-aspartate) glutamate receptors agonist. Glycine accumulation in glycine encephalopathy results in overstimulation of the excitatory NMDA receptors causing seizures (6). The etiology of seizures in metabolic diseases can also be due energy deficiency. Glucose is the main energy source for the brain; therefore, diseases that cause hypoglycemia (e.g., fatty acid oxidation defects) or impair brain glucose transport [e.g., glucose transporter type 1 (GLUT-1) deficiency] can cause brain energy deficiency leading to seizures. Additionally, mitochondrial disorders can result in impaired adenosine triphosphate (ATP) production and seizures (1). Cofactor deficiency is another cause for seizures in certain metabolic diseases. Pyridoxal phosphate is an essential cofactor for several enzymatic reactions including neurotransmitter metabolism. This cofactor is deficient in pyridoxine-dependent epilepsy and pyridoxal phosphate-responsive epilepsy (7). A small number of metabolic diseases are associated with structural brain malformations that can cause seizures. The classic example is polymicrogyria that can be seen in peroxisomal disorders and predisposes to seizures due to disrupting neuronal circuits (8). It is worth mentioning that etiology of seizures in metabolic diseases can be more complex and involves multiple mechanisms (1).

Having underlying metabolic diseases causing seizures should be considered when evaluating children with epilepsy. Generally, it is uncommon to see seizures as an isolated presentation of metabolic diseases; therefore, isolated seizures with no other neurological, systemic, or metabolic features are less likely to be due to metabolic diseases (9). Seizures caused by underlying metabolic diseases (metabolic seizures) should be particularly considered in unexplained neonatal seizures, early myoclonic epileptic encephalopathy, hypsarrhythmia, unexplained hypoxic ischemic encephalopathy, refractory seizures, seizures related to stress, fasting, or food intake, aversion or intolerance to food particularly protein, seizures associated with developmental delay, growth failure, or episodes of vomiting or encephalopathy, family history of epilepsy or unexplained neonatal death, parental consanguinity, and seizures associated with abnormal head size (micro or macrocephaly), coarse or dysmorphic facial features, unusual odor, lens or retinal abnormalities, organomegaly, dermatitis and hair abnormalities, movement disorders, and metabolic derangements including hypoglycemia, metabolic acidosis, hyperammonemia, and hyperlactatemia (1).

Metabolic seizures can be caused by various metabolic disorders including amino acids metabolic disorders, disorders of energy metabolism, cofactor-related metabolic diseases, purine and pyrimidine metabolic diseases, congenital disorders of glycosylation, and lysosomal and peroxisomal disorders (Table 1). In this review article clinical approach to metabolic seizures is presented followed by discussion of metabolic disorders commonly presenting with seizures.

**Table 1:** Metabolic disorders presenting with seizures.

**Amino acids metabolic disorders**
•Serine deficiency •Glycine encephalopathy •Maple syrup urine disease •Urea cycle disorders •Organic acidemia •Sulfite oxidase deficiency •Phenylketonuria
**Disorders of energy metabolism**
•Mitochondrial diseases •Glucose transporter type 1 deficiency •Guanidinoacetate methyltransferase deficiency •Fatty acid oxidation defects •Disorders of gluconeogenesis •Glycogen storage disorders
**Cofactor-related metabolic disorders**
•Pyridoxine-dependent epilepsy •Pyridoxal phosphate-responsive epilepsy •Early-onset vitamin B6-dependent epilepsy •Cerebral folate deficiency •Methylenetetrahydrofolate reductase deficiency •Molybdenum cofactor deficiency •Biotinidase deficiency •Holocarboxylase synthetase deficiency
**Purine and pyrimidine metabolic disorders**
•Lesch-Nyhan syndrome •Adenylosuccinate lyase deficiency •Dihydropyrimidine dehydrogenase deficiency •Dihydropyrimidinase deficiency
**Peroxisomal and lysosomal disorders**
•Zellweger spectrum disorder •Gaucher disease type 2 and 3 •Niemann-Pick type C •Metachromatic leukodystrophy •Neuronal ceroid lipofuscinosis •GM1 and GM2 gangliosidosis •Sialidosis
**Congenital disorders of glycosylation**

## Clinical Approach to Metabolic Seizures

When a newborn, an infant, or a child presents with seizures the clinical approach should include a detailed medical history, physical examination, and a comprehensive evaluation including laboratory tests, electroencephalogram (EEG), and brain imaging when indicated (1).

While taking the history it is very important to take details about diet, growth, development, and episodic symptoms. Children with metabolic diseases may have intolerance or aversion to certain food particularly protein, failure to thrive, developmental delay, or unexplained episodes of encephalopathy or recurrent vomiting. Concerning findings in family history include parental consanguinity and family history of seizures or unexplained neonatal death. In addition, if the seizures are noticed to be related to stress, fasting, or food intake, underlying metabolic diseases should be suspected (1).

In physical examination, significant findings that can raise the suspicion of metabolic seizures include microcephaly (e.g., GLUT-1 deficiency, adenylosuccinate lyase (ADSL) deficiency, and serine biosynthesis defects), macrocephaly (e.g., Tay-Sachs disease), dysmorphic features (e.g., Zellweger syndrome), coarse facial features (e.g., GM1 gangliosidosis), unusual odor (e.g., Maple syrup urine disease), lens dislocation (e.g., sulfite oxidase deficiency and molybdenum cofactor deficiency), cherry-red spot (e.g., GM1 gangliosidosis and GM2 gangliosidosis), hepato(speno)megaly (e.g., Gaucher disease type 2 and 3, GM1 gangliosidosis, and Zellweger syndrome), dermatitis (e.g., biotinidase deficiency and phenylketonuria), sparse hair or alopecia (biotinidase deficiency), and movement disorders (e.g., organic acidemias, guanidinoacetate methyltransferase (GAMT) deficiency, and GLUT-1 deficiency) (1).

Initial laboratory tests usually evaluate common causes including hypoglycemia, electrolyte disturbances, and infections of central nervous system. In addition, blood gases to determine acid-base balance disturbances, ammonia, and lactate should be part of the initial laboratory evaluation. Metabolic derangements can provide some clues for metabolic diseases including hypoglycemia in fatty acid oxidations defects, metabolic acidosis in organic acidemias, and hyperammonemia in urea cycle disorders. Regarding lactate, it is important to know that the seizure itself can increase lactate level but this increase is transient and after seizure resolution lactate is cleared quickly. Therefore, observing a persistent elevation in lactate after 1 or 2 h following a seizure raises the suspicion for metabolic diseases including mitochondrial disorders and organic acidemias (1, 10, 11).

Reviewing newborn screening results can be helpful particularly for neonates presenting with seizures. A second tier of biochemical testing should be ordered based on clinical suspicion made from the presentation and the initial workup. Such tests include plasma amino acids and acylcarnitine profile, and urine organic acids. Specific patterns of abnormalities in these metabolic tests can indicate certain metabolic diseases. Additional metabolic tests can be performed on cerebrospinal fluid (CSF) including glucose, amino acids, and folate, and on urine for purines and pyrimidines, S-sulphocysteine, and guanidinoacetate. Another layer of testing is typically required for diagnosis confirmation including enzyme activity measurement, single gene testing, and functional assays in skin fibroblasts. When the diagnosis remains unclear, additional comprehensive testing can be considered including global metabolomic profiling and whole exome or genome sequencing (1, 12).

Different forms of seizures with variable EEG abnormalities are seen in metabolic seizures. For example, myoclonic seizures are seen in pyridoxine-dependent epilepsy, pyridoxal phosphate-responsive epilepsy, mitochondrial disorders, and glycine encephalopathy; infantile spasms are seen in serine biosynthesis defects, mitochondrial disorders, untreated phenylketonuria, and biotinidase deficiency; and generalized tonic-clonic seizures are seen in GLUT-1 deficiency, mitochondrial diseases, creatine deficiency syndromes, and biotinidase deficiency. Early myoclonic epileptic encephalopathy with burst suppression pattern can be observed pyridoxine-dependent epilepsy, pyridoxal phosphate-responsive epilepsy, glycine encephalopathy, sulfite oxidase deficiency, and molybdenum cofactor deficiency. Hypsarrhythmia can be observed in serine biosynthesis defects and untreated phenylketonuria (1, 13).

Neuroimaging usually shows non-specific changes or no abnormalities in metabolic seizures. However, some changes are more associated with certain metabolic diseases, such as corpus callosum dysgenesis in glycine encephalopathy and pyridoxine-dependent epilepsy, and gyral abnormalities in peroxisomal disorders. Magnetic resonance spectroscopy (MRS) can show elevated lactate peak in mitochondrial disorders, elevated glycine peak in glycine encephalopathy, and decreased creatine peak in creatine deficiency syndromes.

## Amino Acid Metabolic Disorders

Seizures can be observed in various amino acid metabolic disorders including serine deficiency, glycine encephalopathy, maple syrup urine disease, urea cycle disorders, organic acidemia, phenylketonuria, and sulfite oxidase deficiency (Table 1).

### Serine Deficiency

There are three enzymes needed for serine biosynthesis: phosphoglycerate dehydrogenase (PGDH), phosphoserine aminotransferase, and phosphoserine phosphatase. Serine deficiency results from defects in any of these enzymes, most commonly PGDH. Serine has important roles in neuronal function and development. It is needed for protein synthesis and is a precursor for several essential compounds such as phosphatidylserine and sphingomyelin (14).

Serine deficiency is a rare autosomal recessive metabolic disease that has a broad phenotypic spectrum. Neu-Laxova syndrome, which is a lethal multiple malformations syndrome, represents the severe end of serine deficiency spectrum. Less severe forms include the infantile and the childhood presentations. Infantile serine deficiency presents with intrauterine growth retardation, microcephaly, profound developmental delay, hypertonia, congenital cataracts, feeding difficulties, and hypogonadism. Seizures typically start in the first few months of life and include infantile spasms, myoclonic, tonic-clonic, tonic, and atonic. EEG can reveal hypsarrhythmia and multifocal epileptic discharges. Neuroimaging can show hypomyelination, brain atrophy, and hypoplastic cerebellum and pons (15, 16). The childhood form presents with developmental delay and intellectual impairment, behavioral disorders, ataxia, hypertonia, polyneuropathy, and epilepsy including absence and tonic-clonic seizures (15, 17).

Biochemically, plasma and CSF amino analyses typically show low serine and glycine. Serine and glycine plasma levels can be normal in non-fasting state, whereas CSF amino acid analysis is not affected by diet and therefore is more reliable. Diagnosis can be confirmed enzymatically by demonstrating low enzyme activities of serine biosynthesis enzymes in skin fibroblasts or molecularly by identifying mutations in the *PHGDH, PSAT1*, and *PSPH* genes that encode the serine biosynthetic enzymes (15, 16).

Serine and glycine supplements are needed for treatment. In the infantile form, the use of serine and glycine supplementation can improve seizures, irritability, spasticity, and myelination. However, the effect on psychomotor development is minimal. In childhood forms, serine can improve seizure control, school performance, and behavior (15, 17, 18).

### Glycine Encephalopathy

Glycine encephalopathy (non-ketotic hyperglycinemia) is caused by deficiency in the glycine cleavage enzyme leading to glycine accumulation. Glycine is an agonist for the NMDA glutamate receptor. It is also an inhibitory neurotransmitter in brainstem and spinal cord. Overstimulation of NMDA glutamate excitatory receptors results in seizures and the inhibition effect of glycine in brainstem and spinal cord leads to apnea and hypotonia. Glycine encephalopathy is an autosomal recessive metabolic disease with an incidence of 1:60,000 (6).

Glycine encephalopathy can present as severe or attenuated form. The severe form is characterized by lack of developmental progress and intractable seizures, whereas the attenuated form is associated with variable developmental progress and treatable or no seizures. The attenuated form can have three presentations: poor, intermediate, and good. In the attenuated poor presentation developmental quotient (DQ) is <20 and seizures are always present. The attenuated intermediate form is associated with DQ between 20 and 50 and easily treatable or no seizures. Finally, in the attenuated good form the DQ is higher than 50 and there are no seizures. The typical age of presentation is neonatal period or early infancy. Onset during late infancy and childhood can be occasionally observed. In neonatal-onset disease 85% of affected neonates have the severe form and only 15% present with attenuated form. The early infantile presentation between 2 weeks and 3 months is associated with severe form in half of the cases and attenuated form in the other half. Disease onset after the age of 3 months (later onset) is always associated with attenuated form (19).

Neonatal presentation is characterized by progressive lethargy, coma, hypotonia, prolonged apnea that can lead to death without ventilation support, poor feeding, myoclonic jerks, and hiccups. Infantile form presents with developmental delay, hypotonia, and mild or intractable seizures. Late onset form presents with developmental delay and possible mild seizures. Seizures can often be difficult to treat and include myoclonic and generalized seizures. EEG can reveal burst suppression that can progress to hypsarrhythmia or multifocal discharges (6). Neuroimaging can show delayed myelination, corpus callosum agenesis or hypoplasia, and brain atrophy with ventriculomegaly (20).

Biochemically, glycine is elevated in CSF and plasma with high CSF-to-plasma glycine ratio. Diagnosis can be confirmed molecularly by identifying mutations in the *GLDC, AMT*, and *GCSH* genes that encode the glycine cleavage enzyme subunits (6). For treatment sodium benzoate is used to reduce plasma glycine concentrations. NMDA receptor antagonists including dextromethorphan, felbamate, and ketamine can help in seizure control. Severe glycine encephalopathy is associated with poor outcome even with early initiation of treatment. Attenuated forms can be associated with improved outcome with early treatment (6, 21).

### Maple Syrup Urine Disease

Maple syrup urine disease (MSUD) is caused by a deficiency of the branched-chain alpha-ketoacid dehydrogenase (BCKD) enzyme leading to accumulation of branched-chain alpha-ketoacids and the branched-chain amino acids (BCAA) leucine, isoleucine, and valine. Leucine is neurotoxic and its accumulation leads the neurological features observed in this disease. MSUD is an autosomal recessive disease with an estimated incidence of 1:185,000 (22).

Classic MSUD presents during the neonatal period with irritability, poor feeding, lethargy, apnea, opisthotonus, stereotyped movements including fencing and bicycling, coma, ketonuria, and maple syrup odor. Subsequently, acute metabolic decompensation and neurologic deterioration can develop rapidly at any age with infection, surgery, injury, or other stressors. These episodes are usually associated with nausea, vomiting, anorexia, dystonia, ataxia, seizures, brain edema, and altered level of consciousness that may progress to coma and death (23). Besides the classic severe presentation, MSUD can have intermediate and intermittent forms. Intermediate MSUD can present during infancy with feeding difficulties, growth failure, and developmental delay; or later in life with cognitive impairment. Episodes of metabolic intoxication and brain edema can develop with sufficient catabolic stress. On the other hand, intermittent MSUD is typically associated with normal growth and development during infancy and early childhood; however, affected children with this form can develop the clinical features and biochemical derangements of classic MSUD during infections or other catabolic stressors (22).

Biochemically, plasma amino acid analysis reveals elevated alloisoleucine and branched chain amino acids (leucine, isoleucine, and valine) with disturbing the 1:2:3 normal ratio of isoleucine:leucine:valine. Branched-chain keto-acids are detected in urine organic acid analysis. Newborn screening can detect MSUD. The diagnosis is confirmed molecularly by detecting mutations in the *BCKDHA, BCKDHB*, and *DBT* genes that encode BCKD subunits or enzymatically by demonstrating diminished BCKD enzyme activity in cultured fibroblasts, leukocytes, or liver tissue (22). Management of acute presentation includes holding natural protein, BCAA-free amino acids formula with isoleucine and valine supplementation, suppressing catabolism with glucose and insulin infusions and adequate caloric support, treating the underlying cause of the decompensation, and renal replacement therapy when needed. Thiamine trial can be considered. Long-term management includes BCAA-restricted diet with isoleucine and valine supplementation or liver transplantation (22, 23).

### Urea Cycle Disorders

Urea cycle is the main mechanism for clearing nitrogen waste that results from protein turnover. It includes six enzymes: N-acetyl glutamate synthetase (NAGS), carbamoylphosphate synthetase I (CPS), ornithine transcarbamylase (OTC), argininosuccinic acid synthetase (ASS), argininosuccinic acid lyase (ASL), and arginase (ARG). Urea cycle disorders result from defect in any of these six enzymes and lead to the accumulation of ammonia. Ammonia can cause brain damage due to cerebral edema through increased glutamine. Urea cycle disorders are autosomal recessive diseases except OTC deficiency which is inherited in an X-linked manner. The estimated incidence of urea cycle disorders is 1:35,000 (24).

Clinical manifestations depend on the severity of the enzyme deficiency. Severe urea cycle disorders typically present during the newborn period with lethargy, poor feeding, vomiting, irritability, hypotonia, hypothermia, seizures, and coma. Approximately half of the neonates with severe hyperammonemia develop seizures. Hyperventilation leading to respiratory alkalosis is a common early finding and occurs secondary to the effect of ammonia on the brainstem. Hypoventilation and respiratory arrest can occur subsequently because of brain edema causing increased pressure on the brainstem. Mild urea cycle defects occur due to partial enzyme deficiencies. In these disorders, hyperammonemia may be triggered at any time of life by catabolic stress such as illnesses, surgeries, trauma, or prolonged fasting, causing vomiting, loss of appetite, behavioral abnormalities, sleep disorders, lethargy, delusions, hallucinations, psychosis, and brain atrophy (24, 25).

High plasma ammonia is usually the first identified biochemical abnormality. Plasma amino acid analysis is needed to evaluate citrulline and arginine. Citrulline is low in the deficiencies of NAGS, CPS, and OTC, increased in the deficiencies of ASS and ASL, and normal in ARG deficiency. Arginine is markedly elevated in ARG deficiency and low in other urea cycle disorders. Argininosuccinic acid is elevated in ASL deficiency. Urinary orotic acid is markedly elevated in OTC deficiency. The diagnosis is typically confirmed molecularly by identifying mutations in the genes that encode the urea cycle enzymes: *NAGS, OTC, CPS1, ASS1, ASL*, and *ARG1*. If molecular testing is uninformative, enzyme activity for CPS, NAGS, and OTC can be measured in hepatocytes; ASL and ASS in fibroblasts, and ARG in erythrocytes. Newborn screening can defect urea cycle disorders (24).

Acute management aims for prompt correction of hyperammonemia to minimize neurological injury. Hyperammonemia correction can be achieved by decreasing ammonia production from protein intake and breakdown, and ammonia removal. Decreased ammonia production can be achieved by suppressing catabolism through the use of glucose and insulin infusion and increase caloric intake with intralipid administration. Complete protein intake restriction should be limited to 24–48 h, followed by the administration of essential amino acid formula which is important to reverse the catabolic state. Removing ammonia can be achieved by intravenous ammonia-scavenging drugs, sodium benzoate and sodium phenylacetate, along with intravenous arginine hydrochloride (arginine is not used in ARG deficiency). Renal replacement therapy can be considered when ammonia is very high. Hemodialysis is preferred over peritoneal dialysis because it is much more effective in ammonia removal. Long-term management depends on protein-restricted diet with the use of essential amino acids formula, and oral ammonia scavengers. Oral ammonia scavenger medications include sodium benzoate, sodium phenylbutyrate, and glycerol phenylbutyrate. Depending on the urea cycle disorder, arginine or citrulline should be supplemented. Carbamylglutamate is effective for NAGS deficiency and some cases with CPS deficiency. Liver transplantation can be considered for children with severe urea cycle disorders (24, 26).

### Organic Acidemias

Organic acids are intermediate metabolite in several metabolic pathways mostly related to amino acid degradation. Organic acidemias are autosomal recessive disorders that are characterized by the excretion of organic acids in urine. The most common organic acidemias are propionic acidemia and methylmalonic acidemia which are defects in the propionate catabolism pathway involving isoleucine, valine, threonine, methionine, odd chain fatty acids, and cholesterol. Propionic acid is also produced by gut bacteria. Propionic acidemia is caused by a deficiency of propionyl-CoA carboxylase and methylmalonic acidemia is caused by a deficiency of methylmalonyl-CoA mutase. The incidence for propionic acidemia varies between 1:20,000 and 1:130,000 and for methylmalonic acidemia between 1:50,000 and 1:100,000 (27–29).

Propionic and methylmalonic acidemias typically present in the neonatal period with hypotonia, vomiting, poor feeding, lethargy, metabolic acidosis, and hyperammonemia, and without treatment they can progress to coma and death. Milder variants can present at any age with more variable clinical manifestations. Neurological manifestations can include developmental delay, hypotonia, seizures, movement disorders, basal ganglia changes and stroke-like episodes. Other manifestations include neutropenia, thrombocytopenia, pancytopenia, dilated or hypertrophic cardiomyopathy (more commonly with propionic acidemia), chronic kidney disease (with methylmalonic acidemia), and optic neuropathy (27).

Laboratory tests can show high anion gap metabolic acidosis, hypoglycemia, hyperammonemia, hyperglycinemia, elevated transaminases, neutropenia, thrombocytopenia, and pancytopenia. To diagnose organic acidemias, urine organic acid analysis and serum acylcarnitine profile are needed. Urine organic acids analyses reveal elevated 3-hydroxypropionic acid, methylcitric acid, and propionylglycine in propionic acidemia; and elevated methylmalonic and methylcitric acids in methylmalonic acidemia. Plasma acylcarnitine profile reveals elevated propionylcarnitine (C3) in both diseases (27). For propionic acidemia, the diagnosis can be confirmed molecularly by identifying mutations in the *PCCA* or *PCCB* genes that encode propionyl-CoA carboxylase enzyme subunits; and enzymatically by measuring the enzymatic activity of propionyl-CoA carboxylase enzyme (28). For methylmalonic acidemia, the diagnosis can be confirmed molecularly by identifying mutations in the *MMUT* gene that encodes the enzyme methylmalonyl-CoA mutase, and enzymatically by measuring the methylmalonyl-CoA mutase enzyme activity (29). Both conditions can be detected by newborn screening.

Management of acute decompensation includes treating precipitating factors such as infection and dehydration, holding protein intake, administering glucose, lipid, and insulin to suppress catabolism, and administering sodium bicarbonate to correct acidosis and carnitine to enhance organic acids excretion in urine. If these measures fail, hemodialysis needs to be considered. Chronic therapy includes dietary restrictions and oral carnitine supplementation. Restrictions in the amino acids producing propionic acid (isoleucine, valine, methionine, and threonine) is needed for propionic and methylmalonic acidemias. Therapeutic trial of vitamin B12 (adenosylcobalamin), the cofactor for methylmalonyl-CoA mutase, can be considered in methylmalonic acidemia. Therapeutic trial of biotin, the cofactor for propionyl-CoA carboxylase, can be considered in propionic acidemia. Liver transplantation is a consideration in children with poorly controlled disease with frequent metabolic decompensation despite appropriate dietary and pharmacological treatment (27–29).

## Disorders of Energy Metabolism

Energy deficiency can cause seizures in various metabolic disorders including mitochondrial diseases, glucose transporter type 1 (GLUT-1) deficiency, guanidinoacetate methyltransferase (GAMT) deficiency, and disorders causing hypoglycemia including fatty acid oxidation defects, disorders of gluconeogenesis, and glycogen storage disorders (Table 1).

### Mitochondrial Disorders

Mitochondria produce most of cellular energy through oxidative phosphorylation which is performed by electron transport chain (ETC or respiratory chain) complexes. Mitochondria are composed of two bilayer membranes. The inner mitochondrial membrane accommodates the ETC complexes that transfer electrons and generate ATP. The vast majority of mitochondrial proteins are encoded by the nuclear DNA (nDNA) and a small percentage is encoded by the mitochondrial DNA (mtDNA) (30).

Mitochondrial diseases include a large group of disorders that result from dysfunction of the mitochondrial respiratory chain due to defects in mitochondrial proteins caused by mutations in either nDNA or mtDNA. In mitochondrial diseases, mitochondria cannot generate enough energy to meet the demand of various organs. In addition to energy deficiency, mitochondrial dysfunction results in excessive reactive oxygen species (ROS) production, aberrant calcium handling, and apoptosis dysregulation (30, 31). Mitochondrial diseases due to nDNA mutations are inherited in an autosomal recessive or autosomal dominant manner. Mitochondrial diseases due to mtDNA defects are maternally inherited. The incidence of mitochondrial disorders has been estimated as 1:5,000 (30, 32).

Mitochondrial diseases can affect any organ, particularly organs with high energy needs including nervous system, skeletal and cardiac muscles, liver, kidneys, and endocrine system. Most of mitochondrial diseases affect multiple organs; however, some mitochondrial diseases affect a single organ such as non-syndromic hearing loss and Leber hereditary optic neuropathy. Mitochondrial diseases can present at any age from neonatal age to adulthood. Common manifestations of mitochondrial diseases include exercise intolerance and myopathy, cardiomyopathy, optic atrophy, pigmentary retinopathy, ptosis, external ophthalmoplegia, sensorineural deafness, developmental delay and intellectual disability, seizures, ataxia, dementia, spasticity, migraines, stroke-like episodes, and diabetes mellitus. Some mitochondrial diseases have a characteristic cluster of manifestations making discrete clinical syndromes such as MELAS (mitochondrial encephalomyopathy with lactic acidosis and stroke-like episodes), MERRF (myoclonic epilepsy with ragged-red fibers), and NARP (neurogenic weakness with ataxia and retinitis pigmentosa). However, there is often considerable clinical variability, and presentation does not fit into one particular syndrome in many affected individuals (30, 32).

Biochemical screening tests for mitochondrial diseases can reveal lactic acidemia, hypoglycemia, elevated TCA (tricarboxylic acid cycle) intermediates in urine organic acids, and elevated alanine in plasma amino acids. Diagnosis is typically confirmed molecularly by identifying mutations in mtDNA or nDNA genes that encode mitochondrial proteins. With advances in molecular testing, there is less of a need to do tissue biopsies for diagnosis. The histologic examination of affected muscles can reveal ragged red fibers and electron microscopy can show abnormal morphology of mitochondria and mitochondrial proliferation. Histochemical staining for ETC complexes can reveal the severity and heterogeneity of ETC complex deficiencies in affected muscle tissue. Mitochondrial function assessment can be done by measuring ETC complex activities using spectrophotometry on tissues such as skeletal muscle, skin fibroblast, or liver. The assessment of mitochondrial function can also be performed by measuring mitochondrial respiration and glycolysis using the Seahorse instrument (30).

There have been no satisfactory therapies for most mitochondrial diseases. Therapeutic approaches have mostly been symptomatic to provide supportive measures such as physical therapy for hypotonia, hearing aids or cochlear implants for hearing loss, surgical correction for ptosis, and pancreatic enzymes for exocrine pancreatic dysfunction. Exercise is beneficial for mitochondrial disorders. Exercise can improve mitochondrial function and induce mitochondrial proliferation. No specific dietary management has shown consistent benefit for mitochondrial diseases. However, optimizing caloric intake is important to improve oxidative phosphorylation capacity in patients with mitochondrial disorders (33). Several agents have been currently used or studied in mitochondrial diseases. Some agents aim to increase ETC function and modulate oxidative stress such as coenzyme Q10, idebenone, riboflavin, thiamine, lipoic acid, and elamipretide. Other agents function in mitochondrial biogenesis augmentation such as bezafibrate and resveratrol. Arginine and citrulline are used to restore nitric oxide deficiency, and nucleotides are being tried for the restoration of nucleotides pool (33, 34).

### Glucose Transporter Type 1 Deficiency

Glucose transporter type 1 (GLUT-1) transmits glucose to the brain through blood–brain barrier. As glucose is the main source of energy in the brain, defects in this transporter impair brain energy supply. GLUT-1 deficiency is inherited in an autosomal dominant pattern in most cases and in autosomal recessive pattern in some cases. Incidence is estimated at 1:100,000 (35).

GLUT-1 deficiency typically presents with developmental delay, movements disorders, refractory seizures, and microcephaly. Seizures typically start before the age of 2 years and can have variable types including generalized tonic-clonic, complex or simple partial, myoclonic, drop attacks, tonic, absence, and infantile spasms. Most affected children have mixed types. Symptoms can get worse with fasting. EEG obtained while fasting reveals background slow activity with multifocal or generalized high-amplitude spikes. Following a carbohydrate meal, EEG can show a decrease in epileptic discharges (36, 37).

Biochemically, CSF glucose is low with reduced CSF-to-blood glucose ratio. CSF samples need to be obtained during fasting. The diagnosis can be confirmed molecularly by identifying mutations in the *SLC2A1* gene that encode GLUT-1 (37). Management is based on the use of ketogenic diet which provides ketone bodies as an alternative source of energy to the brain. Ketogenic diet can help in seizure control in most affected children; however, the effect on development is less prominent (38).

### Guanidinoacetate Methyltransferase Deficiency

Guanidinoacetate methyltransferase (GAMT) enzyme converts guanidinoacetate to creatine. Impaired GAMT results in the deficiency of creatine which functions as a neurotransmitter modulator and has an essential role in energy storage and transmission. GAMT deficiency is an autosomal recessive disease with an estimated prevalence of 1:500,000 (39).

GAMT deficiency presents with developmental delay and cognitive impairment, with speech and language development being affected more severely. Other features include seizures, movement disorders, and behavioral disturbances including autism and hyperactivity. Seizures occur in more than two-thirds of affected children, have an onset between early infancy and the third year of life, and include generalized tonic-clonic, myoclonic, partial complex seizures, and drop attacks (40).

Biochemically, guanidinoacetate is elevated in blood and urine. MRS can reveal reduced or absent creatine peak. Diagnosis can be confirmed molecularly by identifying mutations in the *GAMT* gene. Treatment aims at the correction of creatine deficiency with creatine-monohydrate supplementation and the reduction of guanidinoacetate accumulation through ornithine supplementation and arginine restriction. The latter is achieved through a protein restricted diet and arginine-free essential amino acid formula (40).

## Cofactor-Related Metabolic Disorders

Seizures are common features of various cofactor metabolic disorders including pyridoxine-dependent epilepsy, pyridoxal phosphate-responsive epilepsy, early-onset vitamin B6-dependent epilepsy, cerebral folate deficiency, methylenetetrahydrofolate reductase (MTHFR) deficiency, molybdenum cofactor deficiency, biotinidase deficiency, and holocarboxylase synthetase deficiency (Table 1).

### Pyridoxine-Dependent Epilepsy

Pyridoxine-dependent epilepsy is caused by antiquitin enzyme deficiency. Antiquitin is a part of lysine metabolism pathway. It functions as piperideine-6-carboxylate (P6C) dehydrogenase and alpha-aminoadipic semialdehyde (αAASA) dehydrogenase. Antiquitin deficiency causes accumulation of αAASA and P6C. P6C can bind and inactivate pyridoxal phosphate, which is an important cofactor for the metabolism of several neurotransmitters. Pyridoxine-dependent epilepsy is an autosomal recessive disease with an estimated prevalence between 1:100,000 and 1:700,000 (7, 41).

Pyridoxine-dependent epilepsy typically presents during the neonatal period with seizures. Atypical presentation can start after neonatal period but before 3 years of age. Seizures are resistant to standard antiepileptic treatment and can be tonic, clonic, or myoclonic. Associated clinical features include respiratory distress, poor feeding, lethargy, irritability, and hypotonia. Neuroimaging occasionally revels corpus callosum agenesis or hypoplasia, ventriculomegaly, and mega cisterna magna. EEG usually shows burst suppression pattern that can evolve into hypsarrhythmia and multifocal or focal discharges (42, 43).

Pyridoxine-dependent epilepsy should be suspected in newborns presenting with early myoclonic epileptic encephalopathy with EEG showing burst suppression pattern. When pyridoxine-dependent epilepsy is suspected, pyridoxine trial is indicated. The diagnosis is established clinically by demonstrating a response to pyridoxine. Intravenous pyridoxine administration with EEG monitoring can result in cessation of seizures with corresponding EEG changes within several minutes. Oral pyridoxine can result in seizure cessation within 3–5 days. Pyridoxine trial should be performed in intensive care units because it might be associated with transient isoelectric EEG, which can be associated with respiratory depression and coma. Biochemically, the diagnosis is supported by the demonstration of elevated αAASA levels in urine, plasma, and CSF; and elevated P6C in urine. Diagnosis can be confirmed molecularly by identifying mutations in the *ALDH7A1* gene that encodes antiquitin (1, 7, 41). Long-term treatment with pyridoxine is required. In addition, lysine-reduction therapies through lysine-restricted diet and competitive inhibition of lysine transport through arginine supplementation, have been recommended to improve outcome. These adjunct therapies can improve biochemical parameters and cognitive development in many affected children (44).

### Pyridoxal Phosphate-Responsive Epilepsy

Pyridoxal phosphate-responsive epilepsy is caused by pyridox(am)ine phosphate oxidase deficiency. This enzyme converts pyridoxine phosphate and pyridoxamine phosphate into pyridoxal phosphate which is the active form of pyridoxine. Pyridoxal phosphate-responsive epilepsy is a very rare autosomal recessive disease (45).

Similar to pyridoxine-dependent epilepsy, pyridoxal phosphate-responsive epilepsy presents with refractory seizures, lethargy, and hypotonia. Preterm delivery with fetal distress can be seen in half of the affected neonates. Seizures appear during neonatal period and include myoclonic, complex partial, tonic-clonic, clonic, tonic, and spasms. Burst suppression is usually observed in EEG (45).

Clinical diagnosis is established by demonstrating seizure cessation with corresponding EEG changes with pyridoxal phosphate administration, usually within an hour. Some affected neonates respond to pyridoxine. Biochemically, diagnosis is supported by demonstrating low pyridoxal phosphate in CSF. Other biochemical abnormalities include elevations of glycine and threonine in CSF and plasma, elevation of 3-methoxytyrosine in CSF, and decrease of 5-hydroxyindolacetic acid and homovanillic acid in CSF. These abnormalities reflect decreased activity of enzymes using pyridoxal phosphate as a cofactor. Diagnosis can be confirmed molecularly by identifying mutations in the *PNPO* gene which encodes pyridox(am)ine phosphate oxidase. Seizures are usually controlled with pyridoxal phosphate (1, 46).

### Early-Onset Vitamin B6-Dependent Epilepsy

Early-onset vitamin B6-dependent epilepsy is a recently described autosomal recessive disorder caused by mutations in the *PLPBP* gene encoding pyridoxal phosphate binding protein, which plays a role in vitamin B6 homeostasis. Affected infants typically present with refractory seizures in their 1st week of life. Other frequent features include intrauterine growth restriction, fetal distress, premature birth, microcephaly, structural brain abnormalities including white matter changes, simplified sulcation, and periventricular cysts, elevated lactate, metabolic acidosis, and elevated glycine in plasma and CSF. Seizure types include generalized tonic-clonic, tonic, clonic, and myoclonic. Burst suppression, multifocal spikes, and focal discharges can be observed in EEG. Affected infants respond to pyridoxine, pyridoxal phosphate, or a combination of both treatments. The majority of patients remained seizure-free whether on pyridoxine or upon switching to pyridoxal phosphate. Diagnosis is molecularly confirmed by identifying mutations in the *PLPBP* gene (47).

### Cerebral Folate Deficiency

Cerebral folate deficiency is caused by defects in the folate receptor alpha which is a major folate transporter across the blood-brain barrier. Defects in this transporter lead to reduced methyltetrahydrofolate (MTHF) levels in CSF. MTHF is a methyl donor which functions in neurotransmitter synthesis and myelin formation. Cerebral folate deficiency is a rare autosomal recessive disease (48).

Cerebral folate deficiency usually presents in early childhood with developmental regression, ataxia, movement disorders, and seizures including myoclonic, tonic, atonic, and generalized tonic-clonic. EEG can show slow background and multifocal epileptiform activity. Neuroimaging can reveal cerebral and cerebellar atrophy and hypomyelination (48, 49).

Diagnosis is based on identifying low MTHF level in CSF with normal plasma folate levels. Diagnosis can be confirmed molecularly by the identification of mutations in the *FOLR1* gene that encodes the folate receptor alpha. Treatment with folinic acid can restore folate CSF levels and improve clinical symptoms (48, 49).

### Methylenetetrahydrofolate Reductase Deficiency

Methylenetetrahydrofolate reductase (MTHFR) catalyzes the reduction of methylenetetrahydrofolate to methyltetrahydrofolate (MTHF). MTHF is also a methyl donor in the conversion of homocysteine to methionine, therefore, MTHFR results in high homocysteine and low methionine. Low methionine leads to a deficiency of S-adenosylmethionine which is a methyl donor for several methylation reactions. MTHFR deficiency is a rare autosomal recessive disease with an estimated incidence of 1:200,000 (50).

MTHFR deficiency typically presents in infancy with lethargy, hypotonia, feeding difficulties, apnea, and seizures including myoclonic, tonic-clonic, and infantile spasms. Seizures can evolve into Lennox-Gastaut syndrome and the disease can progress into developmental regression, coma, and death. Milder forms with later onset can be observed with higher residual enzyme activity and present with gait disturbances and psychiatric disorders. Neuroimaging can reveal delayed myelination and brain atrophy with ventriculomegaly (50–52).

Biochemically, total plasma homocysteine is high, methionine is low, CSF MTHF is very low, and blood folate level can be low. Diagnosis can be confirmed enzymatically by measuring enzyme activity or molecularly by identifying mutations in the *MTHFR* gene. Treatment is based on using oral betaine. Betaine is a substrate for betaine methyltransferase which converts homocysteine to methionine. Therefore, betaine can decrease homocysteine and increases methionine. Outcome can improve with early treatment with betaine (51, 53).

### Molybdenum Cofactor Deficiency and Sulfite Oxidase Deficiency

Molybdenum is a cofactor for the enzymes xanthine oxidase, sulfite oxidase, and aldehyde oxidase. A deficiency of either molybdenum cofactor or sulfite oxidase results in the accumulation of toxic sulfites. Both diseases are rare autosomal recessive diseases. Molybdenum cofactor deficiency and sulfite oxidase deficiency are clinically indistinguishable and present in neonatal period with feeding difficulties, hypotonia, exaggerated startle response, and seizures. Subsequently, spasticity, developmental delay, and lens dislocation appear. Seizures are intractable and EEG can show burst suppression pattern. Neuroimaging can reveal extensive edema with evolution to encephalomalacia with cystic changes and cortical atrophy. Clinical and radiological presentation can mimic hypoxic ischemic encephalopathy (54–56).

Biochemically, S-sulfocysteine is increased in plasma and urine and total homocysteine and cystine are increased in plasma in both conditions. In molybdenum cofactor deficiency serum uric acid level is decreased and urinary xanthine and hypoxanthine levels are increased because of secondary deficiency in xanthine oxidase. Diagnosis of molybdenum cofactor deficiency can be confirmed molecularly by identifying mutations in the *MOCS1, MOCS2*, or *GPHN* genes that encode the enzymes synthesizing molybdenum cofactor. The diagnosis of sulfite oxidase deficiency is confirmed molecularly by the identification of mutations in the *SUOX* gene that encodes sulfite oxidase enzyme (54–56).

No treatment is available for molybdenum cofactor deficiency except for molybdenum cofactor deficiency type A that is caused by defects in *MOCS1*. This type can be treated with intravenous cyclic pyranopterin monophosphate which is a biosynthetic precursor of molybdenum cofactor. However, treatment should be started early before permanent neurologic damage occurs for the treatment to be effective. Similarly, the outcome is poor for isolated sulfite oxide deficiency with no effective treatment. Low cysteine and methionine diet can be beneficial in some children with the mild, late-onset form of the disease (54, 55, 57).

### Biotinidase Deficiency and Holocarboxylase Synthetase Deficiency

Biotin is an important cofactor for several carboxylase enzymes. Holocarboxylase synthetase is the enzyme attaching biotin to the carboxylase enzymes to activate them. Biotinidase is the enzyme recycling biotin. Holocarboxylase synthetase deficiency and biotinidase deficiency are autosomal recessive diseases that are associated with multiple carboxylase enzymes deficiencies. Biotinidase deficiency has an estimated incidence of 1:60,000, whereas holocarboxylase synthetase deficiency is less frequent (58).

Biotinidase deficiency and holocarboxylase synthetase deficiency have overlapping features. Onset of symptoms in holocarboxylase synthetase deficiency is usually before 3 months of age. Holocarboxylase synthetase deficiency typically presents with hypotonia, lethargy, vomiting, hypothermia, and tachypnea associated with metabolic derangements including acidosis and hyperammonemia. The age of onset for profound biotinidase deficiency is variable with an average of 3.5 months of age. Biotinidase deficiency is associated with more neurologic symptoms including hypotonia, ataxia, seizures, developmental delay, and hearing and vision problems. Seizure types include myoclonic, generalized tonic-clonic seizures, and infantile spasms. EEG can reveal sharp multifocal spikes, and hypsarrhythmia. Both holocarboxylase synthetase deficiency and biotinidase deficiency are characterized by alopecia and skin rash (58, 59).

Biochemical derangements include metabolic acidosis, lactic acidemia, and hyperammonemia. Due to multiple carboxylase enzymes deficiencies urine organic acids can reveal elevated 3-methylcrotonylglycine, 3-hydroxyisovalerate, methylcitrate, propionylglycine, and hydroxypropionate. Diagnosis of biotinidase deficiency can be confirmed enzymatically by measuring biotinidase enzyme activity or molecularly by identifying mutations in the *BTD* gene which encodes the biotinidase enzyme. Diagnosis of holocarboxylase synthetase deficiency can be confirmed molecularly by identifying mutations in the *HLCS* gene which encodes the holocarboxylase synthetase enzyme. Biotinidase deficiency and holocarboxylase synthetase deficiency respond to oral biotin with excellent results (58).

## Purine and Pyrimidine Metabolic Disorders

Purines (adenine and guanine) and pyrimidines (thymine, cytosine, and uracil) are nitrogenous bases that are essential components of nucleotides. The addition of pentose monosaccharide (ribose or deoxyribose) to a base results in a nucleoside which can be a ribonucleoside (adenosine, guanosine, cytidine, and uridine) or a deoxyribonucleoside (deoxyadenosine, deoxyguanosine, deoxycytidine, and thymidine). Nucleotides result from the binding of nucleosides to phosphate. The binding of a nucleoside to one, two, or three phosphate groups produces nucleoside mono-, di-, or triphosphate, respectively. Nucleotides are essential for all cells. In addition to their vital role as building blocks for DNA and RNA, they serve as carriers of activated intermediates in the synthesis of a variety of complex molecules, structural components of several essential coenzymes, messengers in signal transduction pathways, regulatory components for many of the metabolic pathways, and currency for energy transfer in cells (60). Seizures can occur in purine and pyrimidine metabolic disorders including Lesch-Nyhan syndrome, adenylosuccinate lyase (ADSL) deficiency, dihydropyrimidine dehydrogenase deficiency, and dihydropyrimidinase deficiency (Table 1).

### Lesch-Nyhan Syndrome

Lesch-Nyhan syndrome is caused by the deficiency of hypoxanthine-guanine phosphoribosyl transferase (HPRT) enzyme which converts hypoxanthine to inosine monophosphate (IMP) and guanine to guanosine monophosphate (GMP). HPRT deficiency results in the accumulation of phosphoribosyl pyrophosphate (PRPP) and depletion of guanine and adenine nucleotides. ATP depletion can result in impairment of energy production, DNA repair, and metabolic defense against oxidant stress. GTP depletion can interfere with the function of G proteins and the formation of tetrahydrobiopterin leading to defects in dopaminergic neurotransmitter system. The accumulation of PRPP leads to an enhancement of the purine synthesis rate and excessive production of uric acid. Lesch-Nyhan syndrome is as X-linked recessive disease with a prevalence of 1:380,000 (60, 61).

Lesch-Nyhan syndrome affects males and is characterized by neurological dysfunction with cognitive impairment, behavioral disturbances, and overproduction of uric acid. Affected boys typically present during the 1st year of life with hypotonia and motor developmental delayed. Abnormal movements appear within the first few years of life. These include dystonia, opisthotonos, choreoathetosis, and pyramidal signs including spasticity, hyperreflexia, and clonus. Most affected children are cognitively impaired in the mild-to-moderate range. Most affected individuals develop self-injurious behaviors between ages 2 and 4 years. These behaviors involve biting of fingers, hands, lips, and cheeks, and banging the head or limbs against hard objects. Other behavioral problems include recurrent vomiting, aggressiveness, impulsiveness, oppositional defiance, and coprolalia. Uric acid overproduction is present from birth and leads to the precipitation of uric acid crystals in the urinary system. Orange crystals typically appear in the diapers. Stones can cause hematuria and urinary tract infections. Uric acid precipitation in joints can cause gouty arthritis. Tophi can also develop. Renal failure is common when uric acid overproduction is not treated. However, renal failure can develop in some individuals even with treatment. Additional features include testicular atrophy with delayed growth and puberty, feeding difficulties, and macrocytic anemia. Approximately one third of affected individuals develop seizures. EEG shows non-specific slowing or disorganization, and neuroimaging often reveals brain atrophy with decreased cerebral volume and caudate nucleus volume (61).

Biochemically, uric acid is elevated in urine and blood. However, normal serum uric acid cannot be used to rule out Lesch-Nyhan syndrome because around 5–10% of affected individuals may have uric acid in the normal or high normal range, particularly before puberty. The diagnosis can be confirmed enzymatically by measuring the HPRT enzyme activity in erythrocytes or skin fibroblasts. Molecularly, the diagnosis can be confirmed by identifying mutations in the *HPRT1* gene (60, 61).

Hyperuricemia is treated with allopurinol which is a xanthine oxidase inhibitor that decreases uric acid production and helps in reducing the risk of nephropathy, gouty arthritis, and tophi. However, controlling serum concentration of uric acid has minimal effect on behavioral and neurological symptoms. Self-injurious and other deleterious behaviors can be managed by physical, behavioral, and medical interventions. To prevent self-injury, all affected individuals require physical restraints. Lip guards and occasionally tooth extraction can be used to avoid self-injury through biting. Partial improvement in self- injurious behaviors can be achieved using both positive and negative conditioning programs. Baclofen or benzodiazepines can improve spasticity, and haloperidol and barbiturates may sometimes improve choreoathetosis. Adequate nutrition should also be provided (60, 61).

### Adenylosuccinate Lyase Deficiency

Adenylosuccinate lyase (ADSL) enzyme catalyzes two steps in purine nucleotide synthesis. ADSL deficiency causes the accumulation of succinylpurines which have toxic effects on the nervous system. ADSL deficiency is a rare autosomal recessive disease.

ADSL deficiency presents in three forms: neonatal fatal, severe, and mild forms. Most affected individuals have the severe form (type I) which is characterized by severe developmental delay, autistic features, feeding difficulty, growth failure, hypotonia, microcephaly, and seizures. Seizures appear in the first few months of life and include partial, myoclonic, tonic, and infantile spasms. Seizures are usually refractory and multiple antiepileptic agents are required. Neuroimaging can reveal hypomyelination, brain atrophy, and cerebellar atrophy particularly of the vermis. The mild form (type II) presents with mild to moderate developmental delay, autistic features, hypotonia, and ataxia. The neonatal fatal form presents with intrauterine growth retardation, severe hypotonia, encephalopathy, intractable seizures, respiratory failure, and early mortality (60, 62, 63).

Biochemically, succinylaminoimidazole carboxamide riboside and succinyladenosine are high in urine, CSF, and plasma. The diagnosis can be confirmed enzymatically by measuring the ADSL enzyme activity in liver, kidney, lymphocytes, or fibroblasts; or molecularly by identifying mutations in the *ADSL* gene. No effective treatment is available (60, 62, 63).

### Dihydropyrimidine Dehydrogenase Deficiency

Dihydropyrimidine dehydrogenase (DPD) catalyzes the conversion of uracil to dihydrouracil and thymine to dihydrothymine. DPD deficiency results in the accumulation of uracil and thymine, and depletion of the end products β-alanine and β-aminoisobutyrate. The reduction of β-alanine, a neuromodulator that can block the reuptake of GABA (gamma aminobutyric acid), can play a role in the development of the neurological symptoms. DPD deficiency is a rare autosomal recessive disease (60).

DPD deficiency is associated with a highly variable phenotype. The disease usually presents in infancy, although later onset may occur. Most affected children display seizures and developmental delay, often accompanied by hypertonia, hyperreflexia, growth retardation, microcephaly, autistic features, ocular abnormalities (microphthalmia, coloboma, nystagmus, and optic atrophy), and cerebral atrophy and white matter abnormalities on neuroimaging. Affected individuals and carrier (heterozygous) adults who receive 5-fluorouracil, a pyrimidine analog used in treatment of various cancers and metabolized by DPD, can develop severe life-threatening toxicity (60, 64).

Biochemically, uracil and thymine are elevated in urine, plasma, and CSF. The diagnosis can be confirmed enzymatically by measuring the DPD enzyme activity in fibroblasts, liver, and blood mononuclear cells. The diagnosis can be confirmed molecularly by the identification of mutations in the *DPYD* gene that encodes the DPD enzyme. Treatment is symptomatic (60, 64).

### Dihydropyrimidinase Deficiency

Dihydropyrimidinase (DHP) catalyzes the cleavage of dihydrouracil into β-ureidopropionate and dihydrothymine into β-ureidoisobutyrate. DHP deficiency results in the accumulation of dihydrouracil and dihydrothymine, and depletion of the end products β-alanine and β-aminoisobutyrate. Similar to DPD deficiency, the reduction of β-alanine can play a role in the development of the neurological symptoms. DHP deficiency is a rare autosomal recessive disease (60, 65).

Similar to DPD deficiency, the clinical picture is very variable. Symptomatic children can present with developmental delay, hypotonia, epilepsy, growth retardation, microcephaly, and white matter abnormalities. Nearly half of the affected individuals present with gastrointestinal manifestations including feeding difficulties, gastroesophageal reflux, cyclic vomiting, and malabsorption. Similar to DHP, affected individuals and carriers (heterozygous) have increased sensitivity to 5-fluorouracil, leading to severe toxicity. Biochemically, urinary dihydrouracil and dihydrothymine are very high. Moderate elevations of uracil and thymine are also found in the urine. The diagnosis can be confirmed enzymatically by measuring the DHP enzyme activity in liver biopsy. The diagnosis can be confirmed molecularly by the identification of mutations in the *DPYS* gene that encodes the DHP enzyme. Treatment is symptomatic (60, 65).

## Peroxisomal and Lysosomal Disorders

Peroxisomes and lysosomes are cellular organelles that perform distinct metabolic functions. Impairment in these organelles can lead to several metabolic disorders (66). Peroxisomal disorders presenting with seizures include Zellweger spectrum disorder and X-linked adrenoleukodystrophy. Lysosomal disorders presenting with seizures include Gaucher disease type 2 and 3, Niemann-Pick type C, metachromatic leukodystrophy, neuronal ceroid lipofuscinosis, sialidosis, and GM1 and GM2 gangliosidosis (Table 1).

### Zellweger Spectrum Disorder

Peroxisomes are involved in the catabolism of very long chain fatty acids (VLCFA; more than 22 carbons) and branched chain fats such as phytanic acid. They are also involved in the synthesis of several substrates including bile acids and plasmalogens. Defects in peroxisomal biogenesis result in Zellweger spectrum disorder which is an autosomal recessive disease with an estimated prevalence of 1:50,000 (66, 67).

Zellweger spectrum disorder has a phenotypic continuum ranging from severe to mild. Severe form, which used to be called Zellweger syndrome, typically presents in neonatal period with severe hypotonia, characteristic facial features, gyral malformations, seizures, feeding difficulties, hepatic dysfunction, renal cysts, and chondrodysplasia punctata. Affected infants usually make no developmental progress and die during the 1st year of life. Intermediate and mild forms which used to be called neonatal adrenoleukodystrophy and infantile Refsum disease, generally present with developmental delays, hypotonia, hearing and visual impairment, and liver dysfunction leading to a vitamin K-responsive coagulopathy and episodes of hemorrhage. Some individuals may develop leukodystrophy leading to regression and ultimately death (67).

Biochemical abnormalities include elevated phytanic acid and VLCFA and low plasmalogens. The diagnosis can be confirmed molecularly by identifying mutations in one of the PEX genes. There is no effective treatment and management is symptomatic (67).

### Gaucher Disease

Gaucher disease is caused by the deficiency of the lysosomal enzyme glucocerebrosidase (glucosylceramidase). It is an autosomal recessive disease with a prevalence ranging from 1:50,000 to 1:100,000. Gaucher disease has three major clinical types: type 1, type 2, and type 3. The manifestations of type 1 include hepatosplenomegaly, anemia and thrombocytopenia, lung disease, and bone disease, without primary central nervous system disease. On the other hand, Gaucher disease types 2 and 3 are characterized by the presence of primary neurological disease. Type 2 starts before the age of 2 years and is associated with limited psychomotor development, rapid progressive course, and death between the ages 2–4 years. Children with Gaucher disease type 3 may have onset before age 2 years, but they typically have a more slowly progressive course, with survival into the third or fourth decade. Neurological manifestations associate with Gaucher disease types 2 and 3 include pyramidal signs (opisthotonus, head retroflexion, spasticity, and trismus), bulbar signs (stridor, squint, and swallowing difficulty), oculomotor involvement (oculomotor apraxia, saccadic initiation failure, and opticokinetic nystagmus), generalized tonic-clonic seizures, progressive myoclonic epilepsy, dementia, ataxia, and cerebral atrophy on neuroimaging (68).

The enzymatic diagnosis is made by demonstrating low glucocerebrosidase enzyme activity and the molecular diagnosis is established by the identification of mutations in the *GBA* gene which includes the glucocerebrosidase enzyme. Although enzyme replacement therapy is very beneficial for Gaucher disease type 1, it has limited benefits in types 2 and 3 as it does not alter the ultimate prognosis of neurological disease in Gaucher (68).

### Niemann-Pick Disease

Niemann-Pick disease type A and type B are caused by acid sphingomyelinase deficiency and manifest with hepatosplenomegaly, developmental delay, failure to thrive, cherry-red spot, and lung and bone disease (69). Niemann-Pick disease type C is caused by a deficit of cholesterol esterification. Niemann-Pick disease type C is an autosomal recessive disease with an estimated prevalence of 1:150,000. It is characterized by visceral and neurological manifestations depending on the age of onset. Infantile presentation is typically associated with hepatosplenomegaly and lung disease. Childhood presentation is associated with hypotonia, developmental delay, ataxia, dysarthria, dysphagia, seizures, and dystonia. The adolescent and adult presentations typically include dementia or psychiatric manifestations. The diagnosis is confirmed molecularly by identifying mutations in *NPC1 or NPC2* genes and management is symptomatic (66, 70).

### Metachromatic Leukodystrophy

Metachromatic leukodystrophy is caused by arylsulfatase A deficiency. It is an autosomal recessive disease with an overall prevalence between 1:40,000 and 1:160,000. The disease typically appears before 30 months of age (late-infantile) presenting with weakness, hypotonia, hyporeflexia, clumsiness, frequent falls, toe walking, and dysarthria. The disease progresses causing regression, spasticity, neuropathy, seizures, and vision and hearing impairment. Neuroimaging reveals leukodystrophy. Less frequently the disease can appear after the age of 30 months (juvenile onset) with decline in school performance, behavioral problems, gait disturbances, and slower progress than the late-infantile form. The disease can also have an adult-onset presentation with declined school or job performance, emotional lability, personality changes, psychosis, and neurological symptoms. Diagnosis is confirmed enzymatically by demonstrating low arylsulfatase A enzyme activity and molecularly by the identification of mutations in the *ARSA* gene which encodes the arylsulfatase A enzyme (71).

## Congenital Disorders of Glycosylation

Congenital disorders of glycosylation (CDG) represent a clinically and genetically heterogeneous group of disorders that are caused by hypoglycosylation of proteins and lipids (72). Seizures are commonly seen in several CDG (73). Although no specific seizure phenotype is linked to CDG, one report suggested that migrating partial seizures should trigger evaluation for CDG especially when there is multisystemic involvement (74). One glycosylation disorder caused by biallelic mutations in the *CAD* gene was recently identified. *CAD* encodes a multifunctional enzyme, cytoplasmic carbamoyl-phosphate synthetase 2, that is involved in *de novo* pyrimidine biosynthesis. This disorder is associated with global developmental delay, epileptic encephalopathy, and anemia with anisopoikilocytosis. Although this disorder is rare, it is worth mentioning since it can respond to uridine supplementation, ameliorating seizures and improving the neurological phenotype (75, 76).

## Conclusions

Metabolic diseases are relatively uncommon causes for pediatric seizures; however, they should always be considered when evaluating children presenting with seizures. Diagnosing metabolic diseases without delay is essential because the immediate initiation of appropriate therapy for many metabolic diseases can prevent or minimize complications; and seizure control can be achieved when these diseases are appropriately treated. Various metabolic disorders can cause seizures including amino acids metabolic disorders, disorders of energy metabolism, cofactor-related metabolic disorders, purine and pyrimidine metabolic disorders, peroxisomal and lysosomal diseases, and congenital disorders of glycosylation.

## Author Contributions

AE-H and MA wrote the initial draft. RA and MM reviewed, edited, and modified the manuscript. All authors contributed to the article and approved the submitted version.

## Conflict of Interest

The authors declare that the research was conducted in the absence of any commercial or financial relationships that could be construed as a potential conflict of interest. The Handling Editor declared a past co-authorship with two of the authors MA and AE-H.
